# Impact of Arterial Stiffness on In-Stent Restenosis in the Era of Drug-Eluting Stents

**DOI:** 10.31083/RCM23847

**Published:** 2025-06-19

**Authors:** Yiquan Huang, Shaozhao Zhang, Xiaomin Ye, Zhuoshan Huang, Zhenyu Xiong, Xiangbin Zhong, Yifen Lin, Menghui Liu, Xiaodong Zhuang, Xinxue Liao

**Affiliations:** ^1^Department of Cardiology, Xiamen Key Laboratory of Cardiac Electrophysiology, Xiamen Institute of Cardiovascular Diseases, The First Affiliated Hospital of Xiamen University, School of Medicine, Xiamen University, Xiamen 361003, China; ^2^Cardiology Department, First Affiliated Hospital of Sun Yat-Sen University, 510080 Guangzhou, Guangdong, China; ^3^NHC Key Laboratory of Assisted Circulation (Sun Yat-Sen University), 510080 Guangzhou, Guangdong, China; ^4^Department of Cardiovascular Medicine, The Third Affiliated Hospital, Sun Yat-sen University, 510080 Guangzhou, Guangdong, China

**Keywords:** arterial stiffness, drug-eluting stents, in-stent restenosis, pulse pressure, pulse pressure index

## Abstract

**Background:**

In the era of drug-eluting stents (DESs), few studies have explored the association between arterial stiffness and the risk of in-stent restenosis (ISR).

**Methods:**

Pulse pressure and pulse pressure index (PPI), which are noninvasive measures of arterial stiffness, were measured before percutaneous coronary interventions (PCI). PPI is the ratio of pulse pressure to systolic blood pressure. ISR was defined based on the angiographic evidence of ≥50% stenosis within the previously stented segment. Logistic regression was used to calculate the odds ratios (ORs) and 95% confidence intervals (CIs) for ISR.

**Results:**

A total of 644 patients were collected, including 72 patients in the ISR group. Pulse pressure and PPI were significantly higher in the ISR group (ISR vs no ISR: pulse pressure, 58.5 ± 16.3 vs 53.1 ± 13.7 mmHg [*p* = 0.002]; PPI, 0.43 ± 0.07 vs 0.40 ± 0.07 [*p* = 0.001]). Multivariable-adjusted ORs for ISR, for tertile3 vs. tertile1, were 2.73 (95% CI, 1.33–5.62; *p* = 0.006) and 2.12 (95% CI, 1.04–4.31; *p* = 0.038) for pulse pressure and PPI, respectively. The ORs for ISR with a 1-standard deviation (SD) increase in pulse pressure and PPI were 1.41 (95% CI, 1.09–1.83; *p* = 0.010) and 1.52 (95% CI, 1.15–2.01; *p* = 0.003), respectively.

**Conclusions:**

Arterial stiffness denoted by high pulse pressure and PPI is a predictive factor for ISR. A pre-PCI wide pulse pressure could potentially serve as a marker of risk, as well as a potential target for future therapies.

**Clinical trial registration:**

ChiCTR2000039901, https://www.chictr.org.cn/showproj.html?proj=51063.

## 1. Introduction

Drug-eluting stents (DESs) have been widely used in percutaneous coronary interventions (PCI) and play an important role in reducing the incidence of in-stent restenosis (ISR) [1]. Yet, albeit reduced, ISR has far from disappeared, even with DESs and continues to remain the principal reason for treatment failure after contemporary coronary stenting [2, 3].

Coronary perfusion occurs predominantly during cardiac diastole. As a result, an aggressive reduction in diastolic blood pressure (DBP) may compromise cardiac perfusion and worsen ischemia in patients with coronary heart disease (CHD) [4, 5, 6]. In addition, elevated systolic blood pressure (SBP) is associated with increased afterload and myocardial energy requirements [7, 8]. Pulse pressure, defined as the difference between SBP and DBP, is a marker for increased arterial stiffness. Arterial stiffness is one of the earliest indicators of increased cardiovascular disease risk and can be considered a good predictor of the development of subclinical cardiovascular dysfunction [9, 10]. Therefore, it is no wonder that wide pulse pressure, the combination of a high SBP and low DBP, significantly increases the risk of adverse cardiac events [11, 12, 13, 14, 15]. However, in the drug-eluting stent era, whether pulse pressure is still a significant predictor of ISR remains unknown. Pulse pressure index (PPI), the ratio of pulse pressure to SBP, also serves as a useful index in predicting cardiovascular events [16, 17, 18]. In this context, the aim of our study was to explore the association between arterial stiffness and the risk of ISR, hypothesizing that wide pulse pressure and high PPI would predict ISR in the era of DESs.

## 2. Method

### 2.1 Study Population

The RED-CARPET registry (REal-world Data of CARdiometabolic ProtEcTion, ChiCTR2000039901) was designed to investigate risk factors, prognostic factors and individualized treatment strategies for patients with CHD. For the present analysis, we identified 837 patients on the RED-CARPET registry from January 2013 to December 2019, who experienced drug-eluting stent implantation in the First Affiliated Hospital of Sun Yat-Sen University and returned to the hospital for coronary angiography at least 6 months after stent implantation. Participants missing data on covariates were excluded (n = 193). Data from the remaining 644 patients were retrospectively analyzed (**Supplementary Fig. 1**).

### 2.2 Measurements of Blood Pressure (BP)

BP measurements were performed by a trained nurse. SBP and DBP were measured with an Omron electronic sphygmomanometer (HEM-7156, Omron Healthcare Co., Ltd., Kyoto, Japan) before drug-eluting stent implantation during the first hospitalization (index procedure). Based on the recorded peripheral SBP and DBP, pulse pressure and PPI were calculated as follows:

Pulse pressure = SBP – DBP; PPI = pulse pressure/SBP

### 2.3 Definition of ISR

ISR was defined based on the angiographic evidence of ≥50% stenosis within the previously stented segment. In our study, the stenosis degree reported by coronary angiography as moderate or moderate-severe (50%–70% of stenosis), severe or above (≥70% of stenosis) were considered as ISR.

### 2.4 Definition of Other Variables 

Hypertension was defined as SBP ≥140 mmHg, DBP ≥90 mmHg, or anti-hypertensive medication use. Diabetes was defined as fasting glucose ≥7.0 mmol/L, non-fasting glucose ≥11.1 mmol/L, anti-diabetic medication use, or self-reported physician diagnosis of diabetes. CHD was defined as the presence of obstruction of ≥50% of the luminal diameter of at least one native vessel on coronary angiography.

### 2.5 Statistical Analysis

Continuous variables were expressed as mean ± standard deviation and compared using a one-way ANOVA. Categorical variables were expressed as a percentage and compared using χ^2^ statistics. Multivariate logistic regression analyses were used to assess the independent correlates of pulse pressure, PPI and ISR. Covariates in the multivariate regression model were age, sex, creatinine, low-density lipoprotein cholesterol (LDL-C), hypertension, diabetes, and total stented length. These covariates were selected as potential confounders either with a *p*-value of less than 0.05 on the univariate analyses or based on previous studies [2, 3, 19, 20]. In addition, we performed subgroup analysis and tested for interactions by age, gender, smoking status, hypertension, diabetes, follow-up time and number of stents implanted.

All statistical analyses were performed using IBM SPSS Statistics version 26.0 (SPSS Inc., Armonk, NY, USA) and a *p*-value < 0.05 was considered as statistically significant.

## 3. Results

A total of 644 patients’ data were collected, including 72 patients (11.2%) in the restenosis group. Mean (standard deviation [SD]) age was 61.9 (10.3) years, and 79% of participants were men. Table 1 shows the baseline characteristics of patients included in our analysis according to the presence or absence of ISR. Pulse pressure, PPI, age, prevalence of diabetes, number of stents and total stented length were significantly higher in patients with ISR while the group without ISR had significantly higher rates of clopidogrel use. Patient characteristics according to different levels of pulse pressure and PPI are shown in **Supplementary Tables 1,2**.

**Table 1.** **Clinical characteristics of patients**.

Characteristics		ISR (n = 72)	No ISR (n = 572)	*p*
Age, years		64.4 (10.7)	61.6 (10.2)	0.030
Male (%)		73.6	79.5	0.245
Current smoking (%)		37.5	41.3	0.546
Current drinking (%)		9.7	20.3	0.057
SBP, mmHg		133.7 (22.9)	130.4 (19.5)	0.182
DBP, mmHg		75.2 (11.9)	77.3 (12.1)	0.166
HDL-C, mmol/L		0.9 (0.2)	1.0 (0.2)	0.093
LDL-C, mmol/L		2.9 (0.7)	2.9 (1.0)	0.632
Triglycerides, mmol/L		1.7 (0.8)	1.9 (1.6)	0.298
Creatinine, umol/L		95.4 (82.5)	93.8 (79.3)	0.869
Diabetes (%)		43.1	30.8	0.035
Hypertension (%)		63.9	63.3	0.920
Pulse pressure, mmHg		58.5 (16.3)	53.1 (13.7)	0.002
PPI		0.43 (0.07)	0.40 (0.07)	0.001
Medical therapy (%)				
	Aspirin	95.8	95.8	0.991
	Ticagrelor	26.4	19.8	0.189
	Clopidogrel	73.6	83.2	0.045
	Statin	97.2	96.5	0.752
	ACEI/ARB	76.4	80.6	0.399
	Beta-blocker	91.7	88.3	0.394
Target vessel, (%)				
	LM	12.5	8.0	0.202
	LAD	72.2	65.2	0.237
	LCA	27.8	23.8	0.455
	RCA	52.8	41.5	0.068
Number of DES (%)				0.009
	1	30.6	42.0	
	2	25.0	30.9	
	≥3	44.4	27.1	
Stented length, mm		44 (28, 84)	36 (20, 59)	0.016

Values are mean ± SD or median (25th, 75th percentiles) for continuous variables. Abbreviations: SD, standard deviation; SBP, systolic blood pressure; DBP, diastolic blood pressure; PPI, pulse pressure index; LDL-C, low-density lipoprotein cholesterol; HDL-C, high-density lipoprotein cholesterol; LM, left main; LAD, left anterior descending coronary artery; LCx, left circumflex coronary artery; RCA, right coronary artery; DES, drug-eluting stents; ISR, in-stent restenosis; ACEI/ARB, angiotensin-converting enzyme inhibitors/angiotensin II receptor blockers.

**Fig. 1.**
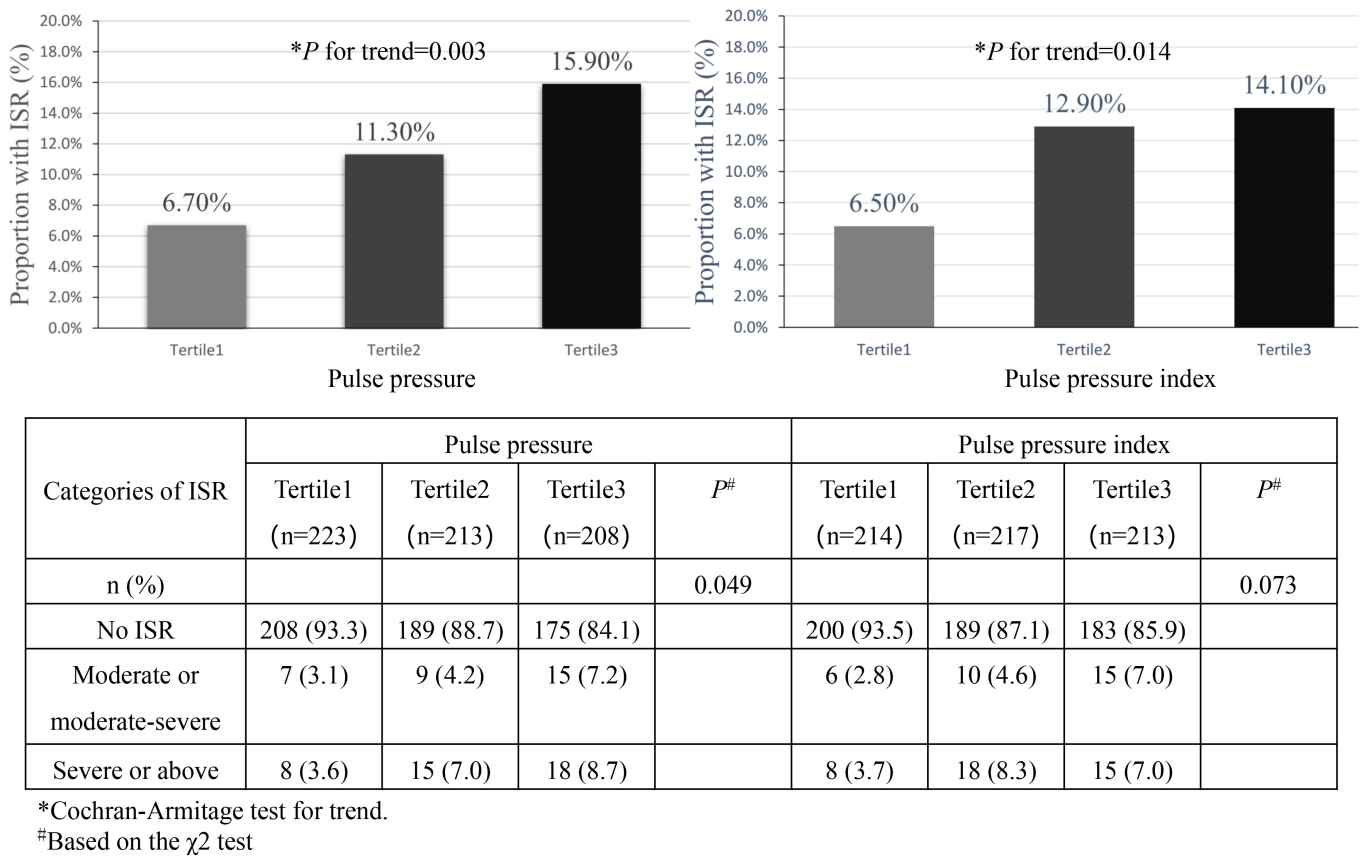
**Number (%) of ISR by pulse pressure and pulse pressure index groups**. Abbreviations: ISR, in-stent restenosis.

Overall, the incidence of ISR was increased with pulse pressure and PPI (Fig. 1). Among different pulse pressure groups, the incidence of ISR from tertile1 to tertile3 was 6.7% (15/223), 11.3% (24/213) and 15.9% (33/208), respectively. A worsening degree of ISR was also associated with a higher pulse pressure (*p* = 0.049) and seemed to be related to a higher PPI (*p* = 0.073) (Fig. 1). By considering pulse pressure, PPI as continuous variables, the OR of restenosis was increased by 41% and 52% when pulse pressure and PPI were increased by 14 mmHg and 0.07 (corresponding to 1 SD), respectively. Results were similar when we categorized individuals by pulse pressure and PPI tertiles and took first tertiles as reference. After adjusting for age, sex, creatinine, LDL-C, hypertension, diabetes, and total stented length, ORs for second and third tertiles were 1.75 (95% CI, 0.87–3.52; *p* = 0.116), 2.73 (95% CI, 1.33–5.62; *p* = 0.006), respectively, for pulse pressure and 2.07 (95% CI, 1.04–4.13; *p* = 0.038), 2.12 (95% CI, 1.04–4.31; *p* = 0.038), respectively, for PPI (Table 2).

**Table 2.** **OR of in-stent restenosis according to pulse pressure and PPI**.

Group	Pulse pressure		PPI	
	OR (95% CI)	p*	OR (95% CI)	p*
Tertile1	1.00 (reference)	-	1.00 (reference)	-
Tertile2	1.75 (0.87, 3.52)	0.116	2.07 (1.04, 4.13)	0.038
Tertile3	2.73 (1.33, 5.62)	0.006	2.12 (1.04, 4.31)	0.038
*p* for trend		0.006		0.048
Per 1 SD	1.41 (1.09, 1.83)	0.010	1.52 (1.15, 2.01)	0.003

*Model are adjusted for age, sex, creatinine, LDL-C, hypertension, diabetes, and total stented length; One SD is14 mmHg for pulse pressure and 0.07 for PPI; Abbreviations: OR, odds ratio; SD, standard deviation; PPI, pulse pressure index; LDL-C, low-density lipoprotein cholesterol; y, years; m, months.

**Fig. 2.**
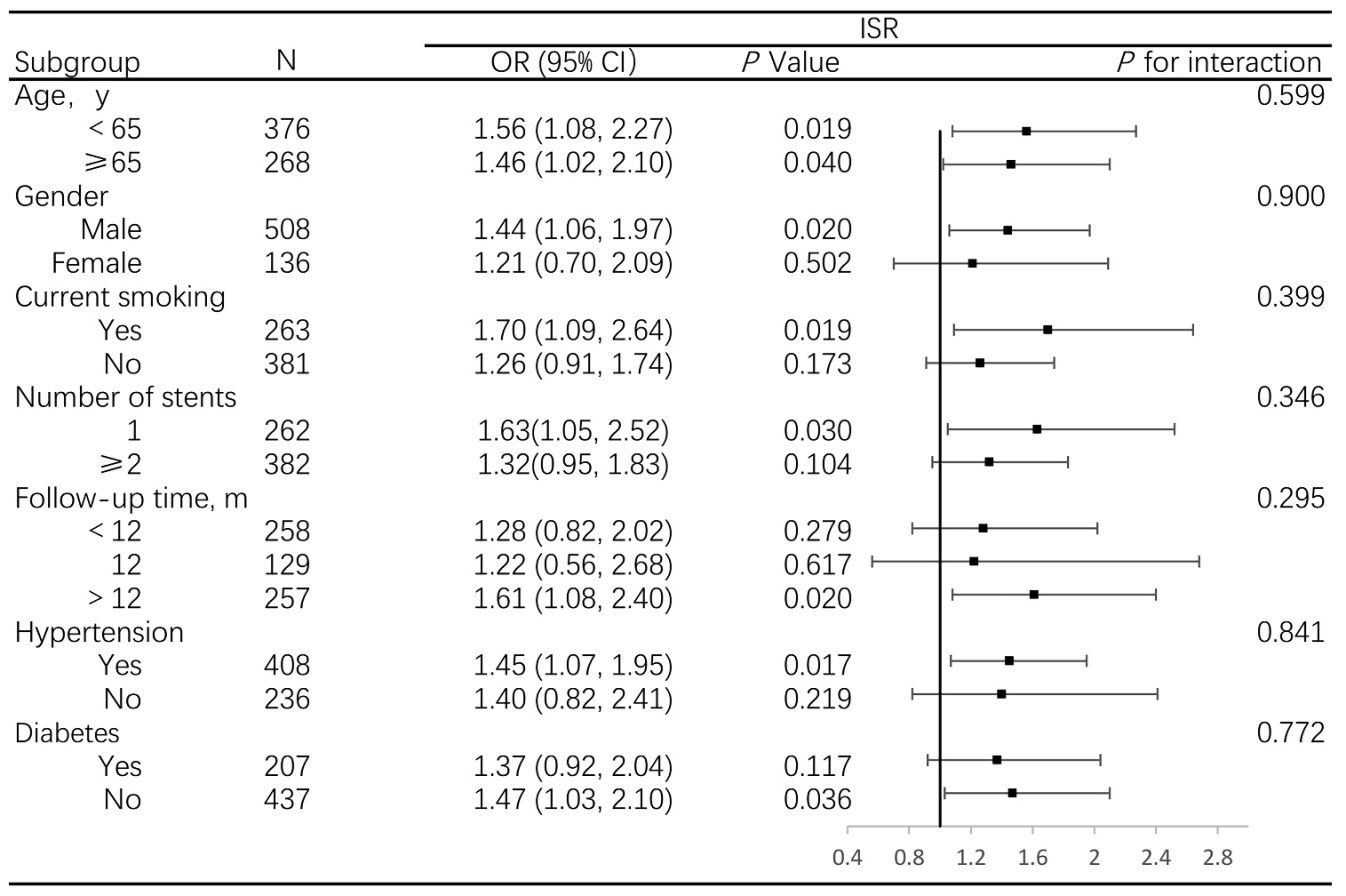
**Association between pulse pressure and ISR, stratified by prespecified subgroups**. Abbreviations: OR, odds ratio; ISR, in-stent restenosis.

When stratified by age, gender, smoking status, hypertension, diabetes, follow-up time and number of stents, the associations between pulse pressure and ISR were stronger in male, smokers, participants with fewer stents implanted and longer follow-up time, hypertension and non-diabetes patients; however, all interactions were not statistically significant (*p* > 0.05 for all interactions, Fig. 2). Similar results of the relationship between PPI and ISR can be seen in **Supplementary Fig. 2**.

## 4. Discussion

To the best of our knowledge, this is the first study to assess the association between arterial stiffness and ISR in the era of DESs. Our study has demonstrated that pulse pressure and PPI are independent predictors of ISR in CHD patients with DESs.

Previous assessments of pulse pressure in relation to restenosis mostly focused on patients after percutaneous transluminal coronary angioplasty (PTCA) and had a small sample size. Nakayama, Y *et al*. [21] found that the pulsatility of the ascending aorta, expressed as PPf (pulse pressure/mean arterial pressure), may predict restenosis 3 months after PTCA in 53 patients. Another study with a sample of 87 patients found that higher pulse pressure was related to an increased risk of restenosis 6 months after PTCA among patients older than 60 years [22]. Retrospective analysis including 84 patients by Jankowski, P *et al*. [23] showed that the risk of restenosis increased by 72% with a 10 mmHg increase in pulse pressure 9 months after PTCA. In the era of drug-eluting stents, few studies have explored the relationship between pulse pressure and the risk of ISR. Our findings were consistent with previous studies, but importantly extended to the era of DESs and included a broader range of patients. Besides, we also did a subgroup analysis and tested for interactions. All interactions were not statistically significant, showing that the association of pulse pressure with ISR was not affected by different subgroups, such as hypertension, diabetes and different follow-up times.

Mechanisms including endothelial injury, thrombosis, proliferation of smooth muscle cells, vascular remodeling, inflammatory reaction, and release of various cytokines may lead to ISR [24, 25, 26, 27]. In short, the ISR process may consist of 4 phases, i.e., platelet aggregation, inflammatory phase, proliferation phase, and late remodeling phase [25, 28]. High SBP is associated with left ventricular hypertrophy, as well as increased afterload wall stress and myocardial oxygen consumption. Besides, low DBP leads to a reduction in coronary perfusion pressure. As a result, a combination of high SBP and low DBP, i.e., wide pulse pressure, is significantly associated with worse cardiovascular outcomes, particularly among those with a history of CHD [15, 29, 30]. Of note, several studies have found that wide pulse pressure resulted in endothelial injury [31, 32, 33], as well as inflammatory response [34, 35, 36, 37]. Thus, wide pulse pressure may contribute to the occurrence and progression of the restenosis process through complex mechanisms, which include endothelial dysfunction and an accelerated inflammatory response.

However, pulse pressure is a dynamic value with two major limitations [16]. First, pulse pressure has alterability in the same individual since BP has large fluctuations in one day. Second, pulse pressure has a “floating” feature in terms of not being relative to the absolute BP level. The pulse pressure may be the same in different individuals with different BP levels. Therefore, PPI was used in our study to overcome the defects of pulse pressure. Our results showed that PPI was also a useful index in clinical evaluation for the assessment of ISR.

## 5. Strengths and Limitations

Our analysis has important strengths, including the prospective design, the extensive and rigorous measurement of covariates, and the rigorous quality control procedures of the individual cohorts. However, this study has several limitations. First, despite the fact that we reduced confounding variables as much as possible, it is unavoidable that residual confounding factors exist. Confounders such as final diameter stenosis, lesion characteristics and body mass index (BMI) weren’t considered in our study. Second, only patients undergoing repeat coronary angiography after previous PCI were included in our study, so it is possible that selection bias derives from the inability to detect clinically silent coronary ISR. Furthermore, blood pressure is dynamic and a single pre-procedural blood pressure may not reflect the patient’s usual blood pressure. It requires further research on the impact of longitudinal pulse pressure, and pulse pressure variability on ISR.

## 6. Conclusions

The present study shows that pulse pressure and PPI independently predict ISR. A wide pulse pressure may serve as a surrogate marker for risk following PCI and represents a potential target for future therapies.

## Availability of Data and Materials

The datasets used and/or analyzed during the current study are available from the corresponding author on reasonable request.

## Supplementary Material

Supplementary material associated with this article can be found, in the online version, at https://doi.org/10.31083/RCM23847.
