# Supplementary material for: Impact of Arterial Stiffness on In-Stent Restenosis in the Era of Drug-Eluting Stents
Source: Rev Cardiovasc Med. 2025 Jun 19;26(6):23847. doi: 10.31083/RCM23847 (PMC12230816; doi:10.31083/RCM23847)
Supplement: Supplementary file 1 [file 2153-8174-26-6-23847-s1.docx]

Supplemental Table 1. Baseline characteristics according to tertiles of pulse pressure.

| Characteristics | Pulse pressure (Tertile1 =223) | Pulse pressure (Tertile2 =213) | Pulse pressure (Tertile3 =208) | P |
| --- | --- | --- | --- | --- |
| Age，years | 57.9 (9.5) | 61.8 (10.4) | 66.2 (9.2) | <0.001 |
| Male (%) | 83.0 | 78.9 | 74.5 | 0.100 |
| Current smoking (%) | 44.4 | 40.4 | 37.5 | 0.146 |
| Current drinking (%) | 19.3 | 23.9 | 13.9 | 0.038 |
| SBP, mmHg | 115.3 (12.9) | 128.3 (11.7) | 150.0 (16.6) | <0.001 |
| DBP, mmHg | 75.4 (12.4) | 76.0 (11.4) | 80.1 (11.9) | <0.001 |
| HDL-C, mmol/l | 0.96 (0.22) | 1.0 (0.24) | 1.0 (0.24) | 0.110 |
| LDL-C, mmol/l | 2.9 (1.0) | 2.9 (0.9) | 2.9 (0.9) | 0.731 |
| Triglycerides, mmol/l | 1.9 (2.0) | 1.7 (1.0) | 1.9 (1.5) | 0.384 |
| Creatinine, umol/l | 83 (73, 96) | 82 (70,95) | 82 (69,99) | 0.207 |
| Diabetes (%) | 26.5 | 34.3 | 36.1 | 0.074 |
| Hypertension (%) | 47.1 | 61.0 | 83.2 | <0.001 |
| Aspirin (%) | 95.1 | 97.2 | 95.2 | 0.472 |
| Clopidogrel (%) | 83.0 | 81.2 | 82.2 | 0.893 |
| Ticagrelor (%) | 21.1 | 21.6 | 18.8 | 0.743 |
| Statin (%) | 96.4 | 96.2 | 97.1 | 0.873 |
| ACEI/ARB (%) | 74.4 | 78.4 | 88.0 | 0.002 |
| Beta-blocker (%) | 91.5 | 87.3 | 87.0 | 0.259 |

Values are mean ± SD or median (25th, 75th percentiles) for continuous variables.

Abbreviations: SD: standard deviation; SBP: systolic blood pressure; DBP: diastolic blood pressure; PPI: pulse pressure index; LDL: low-density lipoprotein; HDL: high-density lipoprotein;

Supplemental Table 2. Characteristics of Participants according to tertiles of PPI.

| Characteristics | PPI  (Tertile1 =214) | PPI  (Tertile2 =217) | PPI  (Tertile3 =213) | P |
| --- | --- | --- | --- | --- |
| Age，years | 57.8 (9.3) | 61.8 (10.3) | 66.0 (9.5) | <0.001 |
| Male (%) | 80.4 | 77.9 | 78.4 | 0.800 |
| Current smoking (%) | 43.0 | 40.6 | 39.0 | 0.470 |
| Current drinking (%) | 20.1 | 23.5 | 13.6 | 0.092 |
| SBP, mmHg | 123.5 (16.3) | 130.5 (19.1) | 138.4 (21.2) | <0.001 |
| DBP, mmHg | 82.2 (11.7) | 77.2 (11.5) | 71.9 (10.7) | <0.001 |
| HDL-C, mmol/l | 0.98 (0.23) | 0.99 (0.23) | 0.99 (0.24) | 0.752 |
| LDL-C, mmol/l | 3.0 (1.0) | 2.9 (0.9) | 2,9 (0.9) | 0.228 |
| Triglycerides, mmol/l | 1.6 (1.1, 2.3) | 1.4 (1.0, 2.0) | 1.6 (1.1, 2.2) | 0.053 |
| Creatinine, umol/l | 82 (73, 94) | 82 (69,96) | 84 (71,100) | 0.200 |
| Diabetes (%) | 32.2 | 28.6 | 35.7 | 0.288 |
| Hypertension (%) | 54.7 | 62.7 | 72.8 | 0.001 |
| Aspirin (%) | 93.9 | 98.6 | 94.8 | 0.036 |
| Clopidogrel (%) | 83.6 | 78.3 | 84.5 | 0.194 |
| Ticagrelor (%) | 19.6 | 23.5 | 18.3 | 0.381 |
| Statin (%) | 96.7 | 96.8 | 96.2 | 0.946 |
| ACEI/ARB (%) | 79.9 | 77.4 | 83.1 | 0.335 |
| Beta-blocker (%) | 93.0 | 87.1 | 85.9 | 0.047 |

Values are mean ± SD or median (25th, 75th percentiles) for continuous variables.

Abbreviations: PPI: pulse pressure index; SD: standard deviation; SBP: systolic blood pressure; DBP: diastolic blood pressure; PPI: pulse pressure index; LDL: low-density lipoprotein; HDL: high-density lipoprotein;


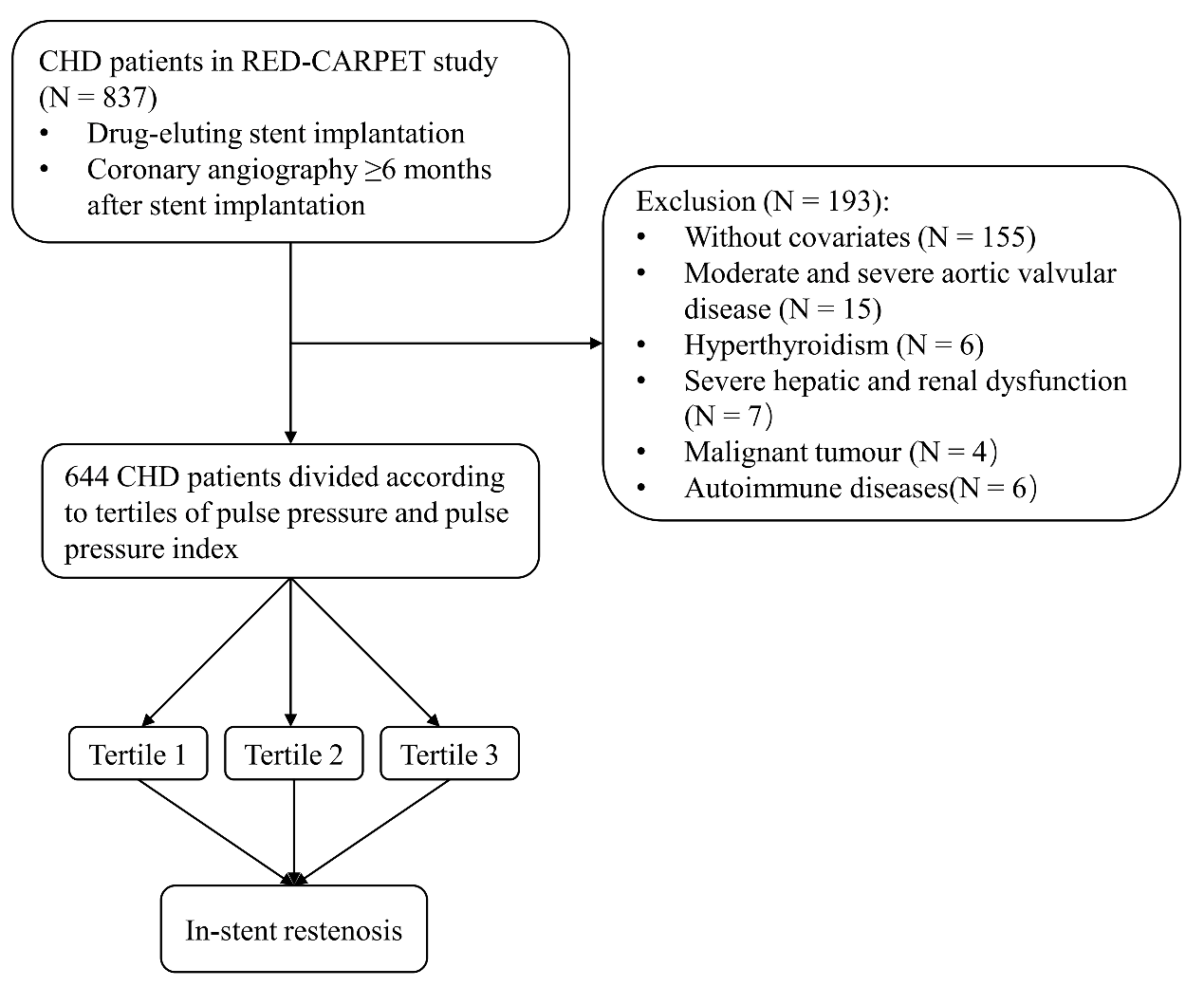


**Supplemental Fig. 1. Study flowchart for selecting patients with drug-eluting stent implantation from RED-CARPET study.** Abbreviations: CHD: coronary heart disease;


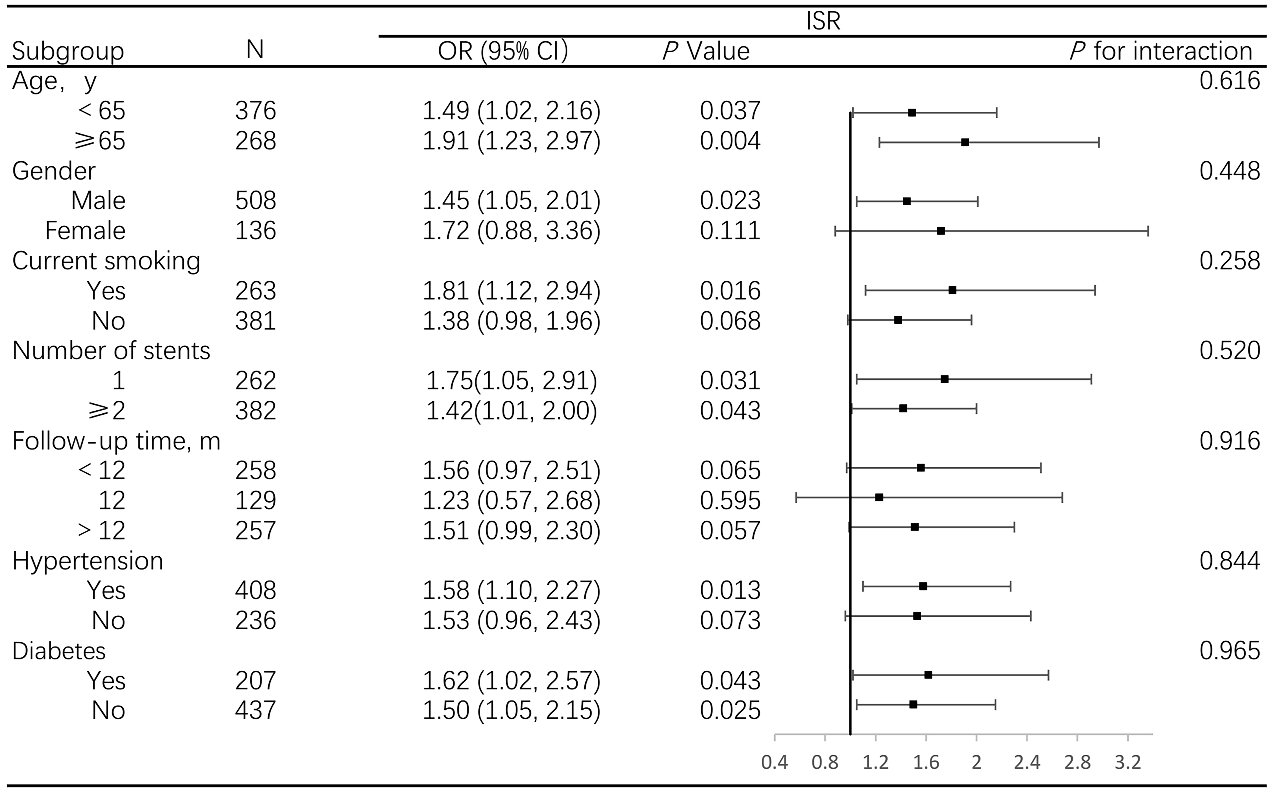


**Supplemental Fig. 2. Association between pulse pressure index and ISR, stratified by prespecified subgroups.** Abbreviations: ISR: in-stent restenosis;
